# The Great Storm at Galveston—From a Medical Standpoint

**Published:** 1900-10

**Authors:** R. H. Harrison


### Correspondence.

### The Great Storm at Galveston—From a Medical Standpoint.

COLUMBUS, TEXAS, September 28, 1900.

*Editor Texas Medical Journal:*

Fortunately, the overwhelming horror which devastated the city of Galveston and a large extent of contiguous coast country on the night of the 8th of September is without a parallel in the history of North America, and rarely in the history of the modern world. The great sea wave which swept the city of Lisbon from its foundation, and destroyed forty thousand lives and hundreds of millions of dollars worth of property nearly a century and a half ago, and the greatest of all known sea waves, which swept the coast of Peru for a distance of two hundred and forty miles, involving some half a dozen cities, and destroying many tens of thousands of lives, are notable instances in which the enormity of the catastrophe excells that of our own recent disaster. In the first of these, the calamity was confined almost entirely to the city of Lisbon, but the destruction of human life and valuable property had not been equaled during the Christian era. In the second, the extent of territory subjected to the devastating influence was immeasurably greater, and the loss of life and destruction of property proportionally larger. In the first, a single city was involved; in the second, a whole empire suffered.

That the West India islands have been subjected to equal if not more overwhelming manifestations of nature's stupendous force can scarcely be doubted. The recent devastation of Porto Rico, with its coincident destruction of life and property, affords an apt illustration of the fact. But these calamitous visitations, unlike those of Lisbon and Peru, are unquestionably the result of terrific atmospheric perturbation; while those of the former places were of undoubted seismic or volcanic origin.

The storm in 1875 in Galveston, and for many miles along the gulf coast, was probably of as great force as the one which has just occurred—possibly of much greater, because it swept a far greater extent of the coast line; and was only less destructive of life and property because the people and property were not there to be destroyed. Its successor in 1885 was less formidable, but still resulting, as in that of 1875, in consequences sufficiently serious to attract our attention as medical men and conservators of the public health. The topography of the city of Galveston is such as to facilitate easy and effective drainage, although its elevation above the sea level is not sufficiently great to afford the amount of current desirable. In addition to this fact there are numerous "dips" throughout the city, and, in fact, all over the island, which prove receptacles for whatever organic matter may be cast up by these occasional floods from the sea, to swelter and decay, poisoning the atmosphere, proving a fruitful source of disease in some of the multifarious forms, it is likely to assume in our semi-tropical climate. Reference to the mortuary reports of the city immediately after these occurrences will attest the fact. The enormous destruction of life, both human and animal, within the city limits, covered, as the remains are, under mountainous piles of wreckage, inaccessible to any available effort to recover or remove promptly, is, therefore, a peculiarly threatening addition to the unsanitary aspects heretofore indicated; but the evil effects of which we may fairly hope to evade by the judicious use of the means which medical progress and sanitary science have provided, and the sympathy of the world has made so promptly available to us.

Let us now return to the consideration of some points in the general features of this great disaster, which have been so vividly portrayed by the daily press and graphically emphasized by the splendid map of the "desolated city" which the enterprise and skill of the *Houston Post* has furnished its readers. A glance at this map shows total destruction of, approximately, two-thirds of the area occupied by the city, and partial destruction of an immense number of public edifices, business houses and higher class residences.

Of the public institutions which suffered damage, the medical profession is more interested in the building of the University School of Medicine, the Sealy Hospital, and last, though not least, St. Mary's Infirmary. The damage to the first of these institutions was mostly confined to the roof of the building, and will be easily repaired in time for the opening of the annual course of medical lectures. The question was sprung during the writer's stay in Galveston as to the propriety of temporary removal of the school to some other place, but wiser counsels prevailed, and it was determined to hurry the repairs, and open the school on the 15th of November.

The damage to Sealy Hospital was also comparatively inconsiderable, and has already been sufficiently repaired to enable that institution to do its full quota of relief work which the stricken city so much needs. The damage to St. Mary's Infirmary was far greater, and will require hundreds of thousands of dollars to make good again. There is, however, enough of the main building left to afford room for the exercise of the devoted charity of the noble people who have it in charge; and relief work is there being accomplished also.

Upon the subsidence of the flood on Sunday morning, September 9th, the scene presented defied description. No pen, nor tongue, nor painter's brush, could convey anything like an adequate conception of it. Death, desolation and dire disaster everywhere. The mangled remains of sea-beaten victims, the floating bodies of women with half-born babes protruding from their bodies, the shrieks and cries of the thousands of victims imprisoned beneath the great mass of wreckage which the waves had piled up in a semicircle around the more populous and substantially built portion of the city, which was only partially ruined—all made a scene of indescribable horror.

It is no wonder, then, that all of humanity that was left to the desolated city stood paralyzed and incapable in the face of the overwhelming disaster. Railway bridges and track, telegraph lines, water craft and every other means of communication had been swept away; and the stricken city stood solitary, helpless and incapable. It was not until the morning of the third day after this dire calamity occurred that an approximation to the reality was flashed across the country, and the nation's magnificent response was made manifest.

The writer, having had some experience in hospital organization and railway surgery, determined at once to offer his services to the stricken city, since it was known that immense numbers of wounded were still living and in need; and it was not known how many members of the local profession had been disabled. Accordingly, he organized a complete hospital outfit from a number of young men from our best families, equipped with all necessary surgical instruments and an outfit for the distillation of water (it having been reported that there was a water famine on the island also). Upon arrival in Houston we were informed that there was no need for any additional aid in Galveston beyond the strictly professional men. Consequently, the young gentlemen who accompanied the writer returned home. In the meantime, New York, Philadelphia and Chicago had organized relief corps of medical men, nurses and every conceivable kind of material that might be needed, was rushed forward in the most abundant profusion. The very extensive destruction of railway lines and water craft made transportation extremely scarce and difficult, on account of which the writer did not reach Galveston until 5:30 on the evening of the 13th. He reported at once to the city health officer, who assured him very promptly that the city did not need any professional assistance. To say that this was a startling announcement is drawing it very mild.

Early the next morning, Friday, the 14th inst., the writer chanced to encounter Dr. Arthur F. Sampson, than whom Texas can boast no more skillful surgeon or accomplished practitioner of medicine. Dr. Sampson had just been appointed chairman of the Fifth Ward relief station, and being already overburdened with the demands of his private practice, was anxious to secure a substitute. He at once requested the writer to take charge of and organize the relief station for him—indicating Fire Station No. 3 as the location. Upon repairing to that point he found a small stock of drugs and dressing material in a little wooden shanty beside the stable for the fire station horses. He soon extemporized an operating table and some shelving for the disposition of the material, and went to work, patients coming in by the score. Dr. John B. Sewell, of Franklin Parish, La.; Dr. A. R. Kuykendall, of Bosque county, Texas; Dr. H. A. Cossett, of the *New York Journal's* staff, and Dr. A. J. Zeilinski, of Houston, were assigned to him as assistants, and the trained nurses Clarence Burgheim, of Houston, and A. C. Rodgers, of the *New York Journal's* staff, also. The demand for attention was so great that we had made some sixty odd dressings before we were able to secure material to keep a record. We made 126 dressings that day, many of the wounds having already become infected, and some of them of considerable gravity. The necessity for hospital accommodation was therefore very evident. This fact becoming known to the Hon. A. J. Rosenthal, of the United States treasury department, he immediately placed all of the spare room in the new custom house at our disposal, and we very speedily established a male and female ward for the accommodation of those whose injuries required it. Miss Elma Hinton, Miss ——— Denny, and Miss Marguerite McKenzie were then added to our list of trained nurses, making our staff complete and effective in every respect.

Of the work accomplished in the six days that it was under the direction of the writer, it is sufficient to say we made five hundred and seventeen dressings, one hundred and twenty-six visits to patients at their residences (eighty-six of which were by Dr. Mercer, another lieutenant of Dr. Sampson's, and one capital operation (by Dr. Sampson)—the only fatal result in our administration.

Of the many dressings made, it is needless to say a large proportion of them were for minor injuries—probably one-third being infected; a very moderate proportion being of considerable gravity. Approximately one-fourth of the cases submitted to our care afforded abundant evidence of the haste with which primary dressings had been made and subsequent neglect to explore the wound and determine its true character. Many wounds that had been closed and stitched together in the primary dressings were found to be filled with foreign matter, such as splinters, fragments of slate, etc., greatly to the detriment of the patient.

The division of labor afforded by the presence of the *New York Journal's* several relief corps, and that from Philadelphia, relieved the demand upon us to such an extent that the city board of health and its auxiliary deemed it wise to discontinue the several relief stations in the city, and have the patients transferred from them to the Sealy Hospital. A resolution to this effect having been passed, the writer handed in his final report.

It remains now to direct attention briefly to the prospective effect of this flood, with all of its horrible incidents, upon the immediate health of the city. The clearing of the streets of wreckage and filth in the business portion of the city, together with the liberal use of powerful disinfectants, has doubtless been of immense value; but it should be remembered that this portion of the city, which contains the majority of the best residences, is surrounded on three of its sides with an immense wall of wreckage, burying beneath it a great mass of organic matter of every conceivable character, in every degree of decomposition, and that the effluvia set free from this putrid mass must necessarily poison the atmosphere, generate disease, and enhance the mortality beyond any recent precedent. The city of Galveston, under normal conditions and an effective sanitary administration, will compare favorably with any city in the Union—as was shown by the administration of Dr. W. C. Fisher, city health officer in 1897-98. But the unsanitary conditions around the city now are herculean in proportion, and in the very nature of things impregnable to rapid removal. The results are already becoming manifest in a rapidly increasing death rate, notwithstanding the diminished population. It is needless, therefore, to say, that the devotion of every energy of the city should be directed to the removal of the seething mass of corruption that now pervades the many streets and alleys in that portion of the city that suffered most severely. The liberal use of disinfectants which have been supplied in such profusion by the humane impulse of our fellow-countrymen should not be neglected. Hastily yours,

R. H. Harrison, M. D.

---

Waco, Texas, 1900.

*Editor Texas Medical Journal:*

The following little episode occurred to break the monotony of the every-day life of two Waco doctors:

Two doctors were recently sitting in their office discussing the merits of some of the newer remedies. Serum therapy, antitoxin, supra-renal capsules, thyroids, etc., were each given due credit according to the experience of one or the other, when suddenly in stepped a hypnotist, with the story of anesthesia and painless amputations under his powerful hypnotic spell.

*"The progressive medical man can not long exist without a thorough knowledge of this branch of the healing art."* The fee was fixed, and the hypnotist went on to explain that it was altogether through repeated suggestion, as "think of sleep," etc., oft repeated, means quiet, undisturbed sleep, induced for any stated time. "Now, you observe that the subject sleeps. The process is so simple that you may imagine yourself faked out of the price of the learning, but I vouch for it; it will stand the test," said he. Then came the doctor's time to try his skill in the new art, but instead of turning to the subject who had yielded so kindly to the suggestion of his instructor, he turned to the instructor, assumed an air of becoming importance, and said, *"Think your fee is paid; think it's paid; it is paid. Go thy way and instruct others in the divine art."* By that time a half dozen individuals had gathered around, and if he had suggested that all join in a most hearty laugh, it could not have been more effectual. The instructor, who appeared to be something of a joker himself, saw the force of the argument, quietly arose from his seat, walked out, and has not been heard of since. It is needless to say that the doctor has faith in the efficacy of the remedy, and believes that his reputation will grow with his daily output of patients.

A Subscriber.

